# Breeding for Resilient Cereals: Integrating Molecular Networks and Trait‐Based Selection to Improve Nitrogen and Water Use Efficiencies

**DOI:** 10.1111/pce.70625

**Published:** 2026-06-02

**Authors:** Grace Achieng Ochieng, Luc Gujer, Shalom Christopher, Annaliese S. Mason, Agim Ballvora

**Affiliations:** ^1^ Department of Plant Breeding, Institute of Crop Science and Resource Conservation (INRES) University of Bonn, Kirschallee 1 Bonn Germany

**Keywords:** cereal crops, genomic breeding, nitrogen use efficiency, root and shoot traits, water use efficiency

## Abstract

Cereal crops are fundamental to global food security, but their production is increasingly challenged by rising demand and the unpredictable impacts of climate change. Among the most limiting and interdependent resources sustaining yields are nitrogen and water. Improving nitrogen and water use efficiencies is therefore essential for sustainable agricultural production. This review examines the relationship between nitrogen use efficiency (NUE) and water use efficiency (WUE) in cereal crops, highlighting how progress in one trait can influence the other. We provide an overview of the physiological and molecular mechanisms that regulate NUE and WUE and discuss how these pathways are interconnected. By comparing key genes across major cereals, we explore how plants balance nitrogen and water acquisition under simultaneous stress. The review further considers below‐ and above‐ground morphological traits and advances in biotechnology aimed at enhancing both efficiencies. We synthesize current knowledge into a conceptual framework for breeding resilient cereal cultivars, including a cross‐tolerance network model and breeding strategies that integrate physiological, molecular, and morphological insights. This review provides a foundation for developing cereals adapted to water‐ and nitrogen‐limited environments.

## Summary statement

This review integrates physiological mechanisms, phenotypic traits, and genomic networks to explain how cereals coordinate nitrogen and water use efficiencies under combined stress, and presents a stress‐based conceptual model to guide breeding for resilience to drought and nitrogen limitation.

## 1 Introduction

The challenge of meeting future food demand is becoming increasingly urgent. By 2050, the world's population is expected to grow so substantially that food production will need to increase by as much as 70 percent (Food and Agriculture Organization of the United Nations. 2023). This growth is compounded by dietary shifts, urbanization, and shrinking arable land, placing unprecedented pressure on agricultural systems. Simultaneously, abiotic stressors such as drought and nutrient limitation rarely act alone, which exacerbates yield losses and undermines food security (Sharma and Bali 2018). Notably, cereal yields in many high‐input regions have plateaued despite continued fertilizer use, pointing to deeper biological constraints (Xin 2022).

In response to these pressures, improving resource use efficiency in crops has become a central goal for both researchers and policymakers. This metric becomes even more critical under co‐limiting conditions, where adaptation to multiple stressors is essential for competitiveness (Hodapp et al. 2019). For instance, a maize plant under drought stress may increase WUE by closing stomata and reallocating assimilates to deeper roots, strategies that are genetically encoded and environmentally responsive (Liu et al. 2023). However, optimizing both NUE and WUE simultaneously is challenging due to the multilayered coordination required between physiological and genetic processes.

The resilience of cereals under simultaneous nitrogen and water limitations is governed by coordinated genetic and molecular networks that regulate nitrogen uptake, transport, and assimilation alongside water‐saving strategies (Hu et al. 2015; Mondal and Molla 2025). Because drought and nitrogen deficiency frequently co‐occur in production environments, understanding how plants integrate these stresses is essential for breeding cultivars that maintain performance under resource scarcity.

To conceptualize the outcomes of concurrent stress exposures, frameworks such as the “Stress Matrix” have been developed to show how plant responses under combined stresses differ from those under individual stress treatments (Gong et al. 2020). This matrix‐based view highlights that interactions between stressors are rarely additive: Crosstalk among hormonal, metabolic, and transcriptional pathways can amplify or moderate plant responses, leading to outcomes that differ from single‐stress conditions (Zhu 2016; Gong et al. 2020). Dual drought–nitrogen stress can also produce adaptive trait shifts. In rice and barley, deeper rooting and narrower root angles improve water capture under drought (Uga et al. 2013), and can incidentally increase access to mobile nutrients such as nitrate that leach to deeper soil layers. However, these benefits are context‐dependent: as drought intensifies, nitrogen deficiency often exacerbates stress by limiting osmotic adjustment, root growth, and photosynthetic capacity (Araus et al. 2020). This reflects a broader trade‐off described in root phenotyping studies (Lynch 2019). Taken together, these interactions show why nitrogen and water limitations cannot be viewed independently. Breeding for improved nitrogen and water use efficiency must account for the way these stresses intersect in real production environments and evaluate genotypes under conditions that reflect these combined pressures.

**Figure 1:**
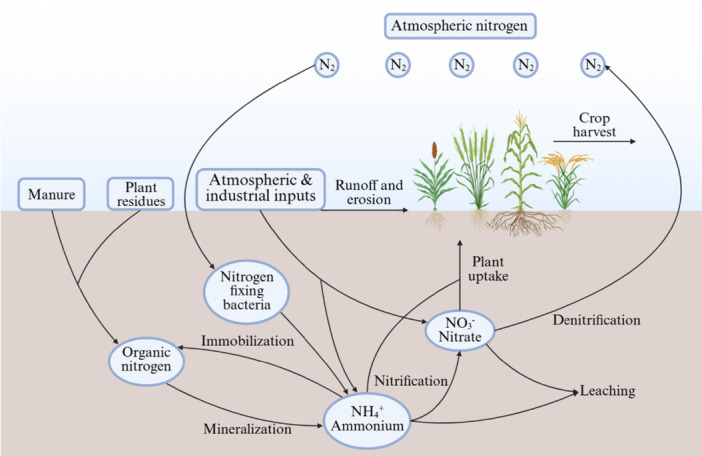
The nitrogen cycle in agroecosystems, showing major input sources and loss pathways relevant to NUE.

Among all the inputs that constrain crop productivity, nitrogen and water remain paramount. The production of synthetic nitrogen fertilizers via the Haber–Bosch process accounts for 1%–2% of global energy use and generates over 350 million tonnes of CO_2_ annually (International Energy Agency 2021). Nitrogen runoff also drives eutrophication and groundwater contamination, making nitrogen use efficiency a critical priority for both productivity and sustainability (Food and Agriculture Organization of the United Nations. 2021). Yet only half of the fertilizer applied each year is absorbed by crops (Ciampitti and Vyn 2014). This inefficiency underscores the urgency for precision approaches to enhance NUE. Nitrogen use efficiency refers to the yield output per unit of nitrogen input, encompassing processes of uptake, translocation, and assimilation (Moll et al. 1982). Figure 1 outlines the nitrogen cycle in agroecosystems, identifying both input sources and major loss pathways. Pinpointing and overcoming these losses through breeding remains a top priority for sustainable intensification.

Water scarcity presents a parallel challenge. Global demand for water is projected to rise by up to 25% in the coming decades, with many cereal‐producing regions already experiencing high variability and persistent shortages (World Resources Institute 2023). Water use efficiency (WUE) is typically expressed as the ratio of biomass or grain yield to water lost via evapotranspiration (Medrano et al. 2015). Water and nitrogen each fulfill indispensable roles in plant growth. Water maintains turgor, regulates temperature, and enables nutrient transport and gas exchange (Knipfer and Fricke 2020; Seleiman et al. 2021), whereas nitrogen is essential for chlorophyll, amino acids, nucleic acids, and photosynthetic enzymes such as Rubisco. The efficiency with which plants acquire and utilize these resources directly determines biomass production and grain yield. Because these processes are physiologically interconnected, improvements in one efficiency trait often influence the other. Evidence supports this interdependence, demonstrating that coordinated improvements in both NUE and WUE outperform single‐trait strategies (Zhang et al. 2024).

Given this context, we explore three central questions in this review: (1) how are nitrogen and water use efficiency regulated in cereals, and what is the nature of their interaction? (2) which shoot and root traits confer tolerance to drought and nitrogen stress in cereals? and (3) what genetic approaches can accelerate the development of cereal varieties resilient to both water and nitrogen limitation? Unlike earlier studies that address these traits separately, our review integrates phenotypic traits, genetic, and breeding perspectives into a unified framework. By synthesizing this knowledge, we aim to support the design of cereal crops that thrive under resource constraints which is an essential step toward climate‐resilient food systems.

### 1.1 The Relationship between Nitrogen and Water Use Efficiencies

The physiological and genetic interplay between nitrogen and water use efficiency has emerged as a critical target for crop improvement, particularly in cereals where resource scarcity and climate variability frequently intersect. Nitrogen and water availability are closely intertwined in plant physiology, and this connection becomes particularly clear under stress conditions. When soils dry, nitrate movement toward the root is reduced because hydraulic flow declines, and key enzymes involved in nitrogen assimilation, such as nitrate reductase, are suppressed (Hu et al. 2015). At the same time, a plant's nitrogen status influences canopy growth, leaf expansion, and stomatal behaviour, which in turn affects transpiration and photosynthesis (Tardieu 2012; Lopes and Reynolds 2010). These processes do not operate independently. Shared hormonal and metabolic pathways, including ABA signaling and source–sink regulation, link water availability to nitrogen acquisition and use. Consequently, when both water and nitrogen are limiting, adjustments in root growth, stomatal conductance, nitrogen remobilization, and carbon assimilation occur simultaneously, often leading to reductions in biomass production and grain quality (Kulkarni et al. 2017; Ahmad et al. 2022).

In this context, WUE reflects how effectively plants convert available water into growth and is modulated by traits such as stomatal conductance, root architecture, and whole‐plant hydraulic capacity. Under drought, stomatal closure reduces water loss but also limits CO_2_ uptake, forcing trade‐offs between conservation and productivity. While drought tolerance and water use efficiency (WUE) are often discussed together, they represent distinct physiological concepts. Drought tolerance reflects a plant's ability to maintain function and survive under water‐limited conditions through mechanisms such as osmotic adjustment, protective metabolite accumulation, and adaptive changes in root system architecture (Tardieu 2012). WUE, in contrast, describes the biomass or grain yield produced per unit of water transpired, and it can be enhanced even under non‐stress conditions through traits that improve carbon assimilation or regulate transpiration (Blum 2009). Importantly, high WUE does not always translate into drought tolerance, particularly when improved WUE results from reduced stomatal conductance. For breeding climate‐resilient cereals, WUE and drought tolerance should therefore be viewed as complementary but non‐substitutable targets (Sinclair 2011).

NUE is shaped by the plant's ability to access soil nitrogen, redistribute it internally, and convert it into structural and functional biomass. Drought commonly reduces nitrogen use efficiency by slowing mineralization and disrupting source–sink dynamics, particularly remobilization to the grain (Plett et al. 2020; Zhang et al. 2022).

Nitrogen and water use efficiencies are governed by largely distinct regulatory pathways, but they intersect at several physiological and transcriptional nodes, including ABA signaling, root system architecture, and stress‐responsive transcription factors, particularly under combined stress conditions. Soil moisture constrains nitrogen mobility, while nitrogen status influences canopy function and gas exchange. As such, physiological integration between water and nitrogen use is crucial, especially under field conditions where both inputs may be limited. Studies increasingly show that simultaneous improvements in NUE and WUE result in greater yield stability than gains in either trait alone (Zhang et al. 2024). This insight has shifted breeding priorities toward identifying genes, traits, and signaling pathways that enable coordinated resource use under stress.

Despite substantial progress, the molecular mechanisms that govern dual N‐ and water‐use efficiency under variable conditions remain incompletely understood. In particular, gene × environment interactions and context‐dependent plasticity present challenges to the development of universally resilient genotypes. Addressing these unknowns is key to advancing trait‐based breeding strategies that can optimize both nitrogen and water use in diverse agricultural settings.

### 1.2 Synergies and Trade‐Offs between Nitrogen and Water Use Efficiency in Cereals

Several studies demonstrate that synergistic gains in both efficiencies are biologically feasible through specific trait combinations and management strategies. For instance, maize hybrids with dense root hairs and high root hydraulic conductance maintained both nitrogen uptake and water retention under deficit irrigation (Kamara et al. 2014). In a diverse panel of bread wheat lines, genotypes with strong osmotic adjustment maintained grain yield and nitrogen remobilization even as soil moisture declined (Duma et al. 2022). These results illustrate that physiological alignment (not necessarily a trade‐off) is possible with the right architecture and signaling capacity. The emergence of such synergies highlights the value of integrative breeding targets: genotypes that coordinate root architecture, hydraulic traits, and nitrogen metabolism in response to environmental cues (Araus et al. 2020). Trait plasticity and genotype × environment interactions play a decisive role in determining whether trade‐offs or synergies emerge in a given context.

When both water and nitrogen are limited, some cereal genotypes exhibit physiological trade‐offs between NUE and WUE. For example, in winter wheat, lines with elevated leaf nitrogen content experienced up to a 15% reduction in WUE under terminal drought. This decline was associated with sustained stomatal opening, which promoted nitrogen assimilation but increased transpiration, leading to greater water loss without proportional biomass gains (Faber et al. 2016). Similarly, crop modeling in maize demonstrated that maximizing nitrogen uptake under drought could accelerate soil moisture depletion, ultimately reducing overall water productivity (Sadras and Rodríguez 2010). These findings underscore the inherent tension between maximizing carbon and nitrogen assimilation when water is scarce.

Trade‐offs between nitrogen and water use efficiency often arise from overlapping regulatory controls at the root–shoot interface. Stomatal conductance, a central determinant of both CO_2_ uptake and transpiration, is influenced by nitrogen availability as well as water status. Hormonal pathways, particularly those involving abscisic acid (ABA), interact with nitrate signaling to modulate this balance, frequently forcing plants to prioritize one resource over the other during stress (Mao et al. 2022; Plett et al. 2020). Characterizing these regulatory conflicts is essential for breeding cereal ideotypes that avoid resource‐use penalties under dual constraint.

Moving forward, the key challenge lies in identifying genetic levers that position cereals within the zone of dual efficiency, rather than forcing compromise between nitrogen and water use. Success in this area will be foundational to breeding climate‐resilient cultivars for future agricultural systems.

### 1.3 Conceptual Framework: Integrating Environment, Plant Traits, Genes, and Genomic Breeding

Improving both nitrogen and water use efficiency in cereals demands an integrative approach that connects environmental stressors, adaptive plant traits, key regulatory genes, and modern breeding strategies. Here, we propose a conceptual framework that traces how external constraints are translated into phenotypic traits and genetic responses, ultimately informing breeding decisions for resource‐efficient cultivars.

**Figure 2:**
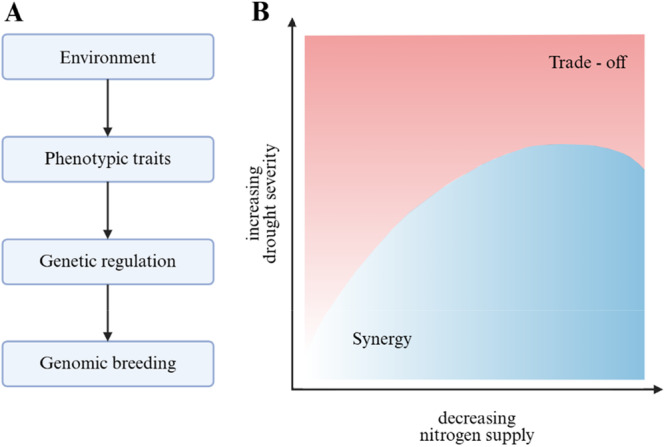
Conceptual framework linking environmental constraints, plant traits, and breeding strategies for nitrogen and water use efficiency in cereals. (A) Hierarchical framework illustrating how environmental conditions are translated into physiological and phenotypic traits, regulatory gene networks, and breeding targets. (B) Drought × nitrogen limitation response space showing regions of synergy and trade‐off between nitrogen and water use efficiencies. Shaded regions indicate functional outcomes: synergy (blue) represents conditions under which coordinated improvements in both nitrogen and water use efficiencies are more feasible, whereas trade‐off (red) represents conditions in which simultaneous optimization becomes more constrained under combined stress. Arrow direction and increasing color intensity indicate increasing nitrogen limitation and increasing drought severity. [Color figure can be viewed at wileyonlinelibrary.com]

Environmental limitations such as drought and nitrogen scarcity trigger adaptive responses in cereals, including deeper rooting and reduced stomatal conductance. In some genotypes, stress adaptation may also be associated with delayed senescence (stay‐green traits), which can help sustain photosynthesis and grain filling under resource limitation. These responses are mediated by gene families that coordinate nitrate transport, water channel activity, and abscisic acid‐activated signaling pathways (Mao et al. 2022; Mondal and Molla 2025). Figure 2A summarizes this hierarchy, illustrating how environmental inputs shape phenotypic traits, regulatory networks, and downstream breeding strategies. Complementing this structural view, Figure 2B depicts how plant responses distribute across regions within a drought × nitrogen limitation gradient. Regions with low to moderate drought and low nitrogen limitation represent conditions of synergy, where improvements in water and nitrogen use efficiency can occur together, as evidenced by coordinated irrigation and nitrogen management in winter wheat (Wang et al. 2025). In contrast, regions characterized by increasing drought severity combined with increasing nitrogen limitation are more likely to reflect trade‐offs, indicating heightened physiological constraints, consistent with findings that water limitation can diminish nitrogen uptake, assimilation, and overall crop performance under stress (Bardehji et al. 2021). Together, these regions emphasize that breeding for resource efficiency must explicitly consider interactions between drought and nitrogen limitation, rather than addressing water and nitrogen responses as independent traits.

As discussed earlier, WUE and drought tolerance are complementary but distinct targets that must both be considered when selecting for performance under water‐limited conditions.

### 1.4 Root–Shoot Interactome and Trait Coordination for Water and Nitrogen Scarcity Stress

Cereal crops cope with water and nitrogen scarcity through coordinated root–shoot adaptations that enhance resource capture, internal allocation, and physiological performance. While these traits arise in response to specific environmental stresses, they are most effective when acting together as whole‐plant strategies. The sections below highlight key root and shoot traits with demonstrated relevance to NUE and WUE in major cereal species. Together, they form a functional basis for selecting ideotypes suited to drought and low‐nitrogen conditions.

## 2 Root Architectural and Anatomical Traits

Effective root architecture enhances soil exploration under limiting conditions. In wheat, rice, and maize, genotypes with deeper and steeper root systems achieve greater access to soil water and nitrogen under drought and low‐input scenarios (Oyiga et al. 2020; Siddiqui et al. 2023; Uga et al. 2013). Traits such as long seminal roots and narrow root angles contribute to deeper soil exploration, which enhances water uptake under drought (Oyiga et al. 2020; Uga et al. 2013). A recent study in wheat further demonstrated that nitrogen‐deficiency tolerance can be conferred through enhanced root system plasticity, including steeper root angles, increased root length, and denser root networks, supported by transcriptome shifts favouring nitrate uptake pathways (Govta et al. 2025). These traits may also improve access to mobile nutrients such as nitrates that tend to leach into deeper soil layers.

Beyond architecture, internal root anatomy plays a critical role in water and nutrient transport. In maize, low‐nitrogen conditions induce wider nodal roots, enhancing xylem conductance by over 20% (Yang et al. 2019). Aerenchyma formation, commonly observed in maize and barley, reduces metabolic cost and promotes deeper soil exploration (Saengwilai et al. 2014). In barley, thinner early‐stage roots aid water conservation, while wider roots at later stages improve nutrient uptake (Oyiga et al. 2020). Additional traits such as high vessel density and optimized cortical thickness support efficient nitrate transport and root penetration in compacted soils (Schneider and Lynch 2020).

### 2.1 Leaf Morphology and Anatomy

Leaf traits mediate the balance between carbon gain and water loss. In maize and wheat, larger flag leaves with dense mesophyll sustain photosynthesis during nitrogen stress (Liu et al. 2023). Reduced stomatal density and smaller stomata improve water regulation under drought in rice and wheat, respectively (Caine et al. 2019). Structural features such as thicker cuticles, enhanced wax deposition, and high minor vein density contribute to leaf hydraulic efficiency and reduce non‐stomatal water loss (Sack and Scoffoni 2013). These above‐ground traits work in tandem with the root system to maintain water status under stress.

Stay‐green refers to the delayed senescence of leaves during reproductive stages, allowing sustained photosynthesis as a consequence of continued nitrogen and water uptake under stress conditions (Koua et al. 2021; Thomas and Ougham 2014). In sorghum, stay‐green alleles help maintain chlorophyll content and photosystem activity during drought, thereby supporting grain filling and yield stability (Borrell and Hammer 2000; Ochieng et al. 2021). Similar benefits have been observed in maize and wheat. Breeding efforts must therefore balance carbon retention with effective nutrient translocation for optimal yield and quality (Thomas and Ougham 2014).

### 2.2 Root–Shoot Interaction

A higher root‐to‐shoot ratio, often observed in barley and wheat under stress, reflects a shift in biomass allocation toward nutrient and water capture (Tolley and Mohammadi 2020). This plastic adjustment is modulated by hormonal feedback and environmental cues. By dynamically adjusting root–shoot balance, cereals improve resource acquisition while maintaining growth efficiency. Together, root and shoot traits form an integrated system that enables cereals to cope with nitrogen and water scarcity. Deep roots support uptake, while leaf traits like stomatal control and delayed senescence improve resource use. Focusing on these combined phenotypes can accelerate breeding for stress‐resilient, efficient crops.

### 2.3 Genetics of Nitrogen Use Efficiency in Cereals

Nitrogen use efficiency is a complex trait controlled by a network of genes involved in nitrogen uptake, transport, assimilation, and remobilization. Deciphering its genetic architecture is essential for breeding cereal cultivars that sustain high yields with reduced fertilizer inputs. NUE is commonly divided into two components: nitrogen uptake efficiency, which reflects a plant's ability to extract nitrogen from the soil, and nitrogen utilization efficiency, which describes how effectively the absorbed nitrogen is converted into biomass and grain yield (Congreves et al. 2015; Moll et al. 1982). Studies have identified key contributors to these processes, including nitrate transporters, assimilation enzymes, and transcriptional regulators.

Nitrate uptake in cereals is primarily mediated by two transporter families: the low‐affinity *NRT1/PTR* (also called NPF) family and the high‐affinity *NRT2* family. These systems allow plants to absorb nitrogen across a wide range of concentrations and adapt to fluctuating conditions. Nitric oxide signalling has also been implicated in the regulation of nitrogen uptake, as overexpression of Phytoglobin1 in rice enhanced expression of high‐affinity nitrate transporters (*NRT2.1, NRT2.3*, and *NRT2.4*) and improved nitrogen accumulation under low‐nitrogen conditions (Samant et al. 2025). In rice, overexpression of the low‐affinity transporter *OsNPF2.2* enhances root‐to‐shoot nitrate translocation and increases grain nitrogen efficiency by up to 15% under paddy field conditions (Li et al. 2015). Allelic variation at *NRT1.1B* (*OsNPF6.5*) between indica and japonica subspecies affects nitrate uptake and NUE, with the indica allele conferring superior performance (Hu et al. 2015). In wheat and barley, homologs of *NPF2.12* are associated with deeper rooting and enhanced nitrate acquisition, contributing to higher grain protein content (Siddiqui et al. 2023). In addition, *TaNAM‐6A* has recently been shown to directly activate nitrate transporter genes such as *TaNRT1.1* and *TaNPF5.5*, thereby promoting nitrogen remobilisation from senescing leaves to developing grains and enhancing grain protein content in wheat (Meng et al. 2024). In sorghum, variation in SbNPF6.4 and *SbNRT2.5* correlates with shoot nitrate content and nitrogen use performance (Morris et al. 2013).

Once absorbed, nitrate is converted into organic nitrogen through the glutamine synthetase/glutamate synthase (GS/GOGAT) cycle, a central pathway in nitrogen utilization efficiency (Congreves et al. 2015; Tabuchi et al. 2005). In maize, *ZmGS3* regulates nitrogen remobilization during grain filling, ensuring efficient transfer to developing kernels (Li et al. 2010). These enzymes are sensitive to external nitrogen supply and environmental cues, allowing cereals to adjust assimilation efficiency in real time. Transcription factors (TFs) coordinate nitrogen signaling with morphological and metabolic processes. In foxtail millet, nitrogen‐responsive TF families such as NAC, Dof, and WRKY modulate root development and nitrogen metabolism under low‐N conditions (Li et al. 2025). These regulatory systems provide cereals with the flexibility to modulate nitrogen acquisition and usage in response to changing soil and climatic conditions.

The expanding genetic knowledge of NUE offers actionable targets for breeding programs. Transporter genes, assimilation enzymes, and transcriptional regulators form a functional toolkit that can be deployed through marker‐assisted selection and genomic breeding. Prioritizing beneficial alleles for nitrogen acquisition and conversion enables the development of cultivars that maintain performance under reduced nitrogen inputs.

### 2.4 Genetics of Water Use Efficiency in Cereal Plants

WUE in cereals is shaped by a network of genes that control water uptake, transport, and conservation under stress. Aquaporins, especially plasma membrane intrinsic protein (PIP) and tonoplast intrinsic protein (TIP) families, control how water moves through plant tissues, shaping both root water uptake and leaf hydration (Maurel et al. 2015; Zhou et al. 2024). Their activity directly influences root hydraulic conductivity and leaf water status. In rice and maize, genes such as *OsPIP1;1* and *ZmPIP2;5* improve water transport efficiency, especially during drought stress (Maurel et al. 2015; Zhou et al. 2024). These genes are developmentally regulated and responsive to environmental signals, allowing plants to fine‐tune water movement based on moisture availability.

Stomata regulate the trade‐off between water loss and CO_2_ uptake. Their formation and spacing is controlled by conserved transcription factors, including SPCH, MUTE, and FAMA, which orchestrate developmental progression in cereals. Under partial root‐zone drying conditions, intrinsic water‐use efficiency was improved through a decoupling of stomatal and mesophyll conductance, allowing photosynthesis to be maintained despite reduced stomatal aperture (Hu et al. 2024). Signaling peptides such as EPF1/EPF2 and receptor kinases like ERECTA further modulate stomatal density and distribution Zhang et al. 2018). These factors collectively influence transpiration and carbon assimilation.

Abscisic acid (ABA) signaling plays a key role in water conservation under drought. It enhances water use efficiency (WUE) by triggering stomatal closure and stress‐responsive gene expression to limit water loss. In cereals, ABA receptors such as *TaPYL1‐1B* in wheat and *OsPYL* genes in rice initiate signaling cascades that regulate ion transport and transcription under stress (Mao et al. 2022).

Together, these mechanisms determine how efficiently a plant uses water throughout its lifecycle. Identifying and stacking favorable alleles across these pathways can accelerate breeding for drought‐resilient, water‐efficient cultivars.

## 3 Synergistic Genetic Mechanisms Underlying Combined Water and Nitrogen Use Efficiency Enhancement

NUE and WUE are often studied in isolation, yet accumulating genetic evidence reveals shared regulatory mechanisms that allow plants to optimize both under stress. Integrating these insights is vital for breeding cereals that maintain productivity under combined nutrient and water constraints.

Abscisic acid (ABA) plays a central role in synchronizing drought and nitrogen responses. ABA receptors such as *TaPYL1‐1B* regulate stomatal closure and root hydraulics while influencing nitrogen uptake (Mao et al. 2022). Nitrate signaling via NRT transporters interacts with ABA pathways, coordinating nutrient uptake with water status (Jia et al. 2023). Plants with disrupted ABA signaling often show imbalances in both water and nitrogen regulation, highlighting their interdependence.

### 3.1 Regulatory Network Hubs: *NAC, DREB*, and *bZIP* Transcription Factors Reveal the Mechanistic Basis of Dual N and Water Use Efficiency

Master regulators like *NAC, DREB, and bZIP* transcription factors mediate adaptive responses across traits. For example, *ZmNAC111* in maize modulates root traits and stomatal conductance under combined stress (Mondal and Molla 2025). *DREB1C* in rice regulates both aquaporins and nitrate transporters, linking water transport to nitrogen assimilation (Mao et al. 2017). Other genes like *OsDREB1F* and *OsNAC5* further illustrate this integrative regulation, contributing to both water retention and nutrient uptake. These hubs coordinate gene networks that integrate environmental cues to optimize efficiency. A broad inventory of genes across species have been identified to play putatively joint roles in NUE and WUE (Supporting Table S1). These genes provide a cross‐species resource for marker‐assisted breeding and genomic selection. Together, these synergistic genes and regulators offer a roadmap for designing cereal cultivars resilient to dual resource stress. Representative examples are summarized in Table 1.

**Table 1:** Representative genes and pathways associated with nitrogen use efficiency and water use efficiency, across cereal species.

Gene	Species	Trait	Locus/accession ID	Function	Reference
*OsNRT1.1B*	*Oryza sativa*	NUE	LOC_Os12g44360	Nitrate transporter	Fan et al. (2016)
*OsGS1;1*	*Oryza sativa*	NUE	LOC_Os03g27210	Cytosolic glutamine synthetase; N assimilation	Tabuchi et al. (2005)
*TaNAC2‐5A*	*Triticum aestivum*	NUE	TraesCS5A02G093600	NAC TF enhancing nitrate uptake & NUE	He et al. (2015)
*NLP7*	Arabidopsis thaliana (translational relevance)	NUE	AT4G24020	Master transcriptional regulator of nitrate signaling	Marchive et al. (2013)
*TaPYL1‐1B*	*Triticum aestivum*	WUE/NUE	TraesCS1B02G148500	ABA receptor improving drought/WUE and N remobilization	Mao et al. (2022)
*OsDREB1C*	Oryza sativa	WUE/NUE	LOC_Os09g35010	AP2/ERF TF controlling stomatal behavior & N–drought responses	Mondal and Molla (2025)
*OsDREB1F*	*Oryza sativa*	WUE/NUE	LOC_Os01g73770	TF improving drought and low‐N tolerance	Zhang et al. (2009)
*OsNAC5*	Oryza sativa	WUE/NUE	LOC_Os11g08210	NAC TF promoting root growth under stress	Jeong et al., 2010
*ZmNAC111*	*Zea mays*	WUE/NUE	GRMZM2G137943	NAC TF regulating drought–nitrogen cross‐talk	Faber et al. (2016)
*ZmTIP2;2*	*Zea mays*	WUE/NUE	GRMZM2G066303	Aquaporin regulating hydraulic–nitrogen coupling	Maurel et al. (2015)
*HvPIP2;1*	*Hordeum vulgare*	WUE	MLOC_57536	Drought/osmotic stress‐responsive aquaporin	Hove et al. (2015)
*OsHKT1;5*	*Oryza sativa*	WUE	LOC_Os01g20160	Na^+^ transporter reducing ionic stress under drought	Munns et al. (2012)

### 3.2 Evolutionary Conservation of Nitrogen and Water Related Gene Families Across Cereals

To examine how key nitrogen‐ and water‐related genes are distributed across cereal genomes, we compiled their annotated chromosomal locations in rice, maize, sorghum, barley, and wheat. Because these genomes differ substantially in size, ploidy, and evolutionary history, cross‐species positional comparisons were interpreted using published cereal synteny as a general framework rather than through a de novo collinearity analysis. Where fine‐scale synteny information was unavailable, we considered broader chromosome correspondence across cereals based on established chromosome‐group relationships.

**Figure 3:**
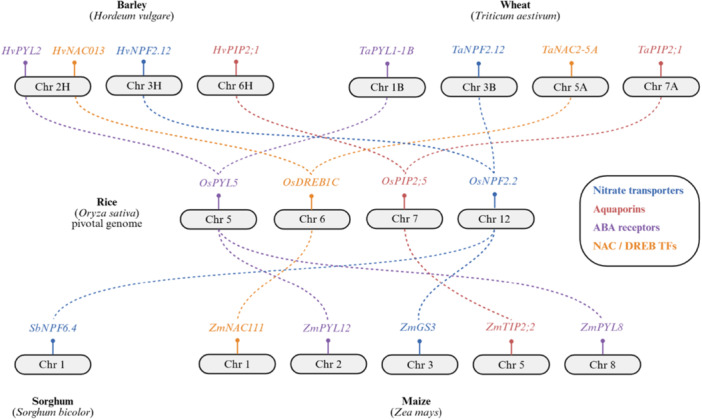
Comparative chromosomal distribution of selected gene families involved in nitrogen and water regulation across major cereal crops, with rice shown as the pivot genome. Dashed lines indicate representative homologous relationships across species inferred from genome annotation, sequence homology, and published functional evidence. Positional comparisons are interpreted in the context of published cereal synteny and broad chromosome‐group correspondence where available, rather than as a de novo collinearity analysis or fine‐scale synteny map. Os, *Oryza sativa*; Zm, *Zea mays*; Sb, *Sorghum bicolor*; Hv, *Hordeum vulgare*; Ta, *Triticum aestivum*. [Color figure can be viewed at wileyonlinelibrary.com]

Our comparison focused on gene families with important roles in nitrogen uptake, water transport, and stress signaling, including nitrate transporters (NPF/NRT), aquaporins (PIP/TIP), ABA receptors (PYL), and NAC/DREB transcription factors. Members of these families are found across the cereal species examined, which suggests that the core molecular systems involved in nitrogen and water responses are widely conserved. At the same time, their distribution across different chromosomes and genomic backgrounds also points to diversification during cereal evolution. In some cases, published cereal synteny provides additional context for these comparisons. For example, *TaNPF2.12* in wheat and *HvNPF2.12* in barley are located in broadly corresponding Triticeae chromosome groups, with barley chromosome 3H corresponding to wheat homoeologous group 3 chromosomes. This supports their interpretation as functionally related homologs within a conserved genomic context. Similar broad chromosome correspondence was considered for other gene families where published information was available (Figure 3).

## 4 Stress Cross‐Tolerance Network Model

**Figure 4:**
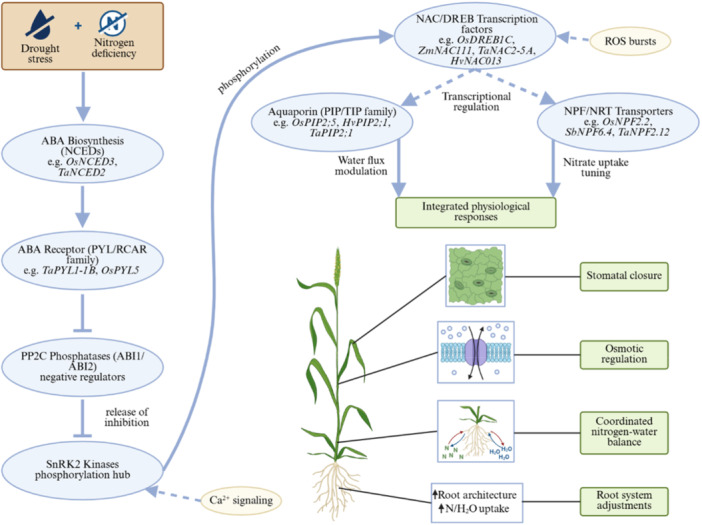
A cross‐tolerance signaling model showing how cereals coordinate nitrogen uptake and water‐saving responses under combined drought and nitrogen stress. The model summarizes key elements of the ABA core module and downstream regulatory nodes to illustrate potential points of coordination between water‐ and nitrogen‐related pathways under combined stress. The schematic is based primarily on insights from (Mao et al. 2022; Mondal and Molla 2025) and related work described in previous sections of this review. [Color figure can be viewed at wileyonlinelibrary.com]

To illustrate how cereals manage simultaneous drought and nitrogen stress, we developed a cross‐tolerance network model (Figure 4 **)** that integrates key regulatory genes across rice, maize, wheat, and barley. The model combines well‐characterized genes such as *DREB1C* in rice and *ZmNAC111* in maize, alongside wheat genes like *TaPYL1‐1B* and *TaNPF2.12*, and their barley counterparts, including *HvNPF2.12* and NAC family transcription factors. The stress model also reflects broader gene families highlighted in Table 1. *NPF/NRT* transporters, PIP/TIP aquaporins, PYL ABA receptors, and NAC/DREB transcription factors without implying that all members of these families have been experimentally validated under combined stress. By organizing these genes into a unified signaling network, the model captures both conserved and species‐specific strategies for coordinating water conservation and nitrogen uptake at the molecular level. Central to this network are genes involved in abscisic acid (ABA) biosynthesis, particularly members of the NCED family which initiate drought signaling by activating ABA receptors such as *TaPYL1‐1B* in wheat. Once ABA binds these receptors, they inhibit PP2C phosphatases, a well‐established switch that lifts repression on SnRK2 kinases (Cutler et al. 2010). This release allows SnRK2 proteins to activate, after which they phosphorylate downstream transcription factors and ion channels that drive drought‐responsive changes in gene expression and stomatal behavior (Soma et al. 2021). These ABA‐dependent steps form the core of the model and link directly to the transcriptional hubs that follow. These receptors, and their orthologs in barley and other cereals, regulate downstream response hubs including *DREB1C, ZmNAC111, TaNPF2.12*, and *HvNPF2.12*. Evidence from cereals supports parts of this model. For example, *TaNAC2‐5A* directly binds the promoters of nitrate‐related genes and enhances nitrate uptake and nitrogen use efficiency in wheat (He et al. 2015; Jing et al. 2024). Similarly, *HvPIP2;1* in barley has been shown to facilitate water and CO_2_ transport and responds to osmotic and salt stress in ways that align with ABA‐linked hydraulic adjustments (Hove et al. 2015).

However, direct regulation of cereal NPF/NRT transporters through an ABA‐activated SnRK2 → NAC/DREB cascade under combined drought and nitrogen stress has not yet been demonstrated. Likewise, connections between Ca^2+^ or ROS signaling, aquaporin activity, and nitrate transport remain largely speculative and likely species‐ or context‐dependent. For this reason, the links shown in Figure 4 should be viewed as working hypotheses that bring together findings from separate studies rather than as fully validated pathways under dual stress conditions. Together, these hubs coordinate nitrate uptake through NPF transporters and modulate water loss via aquaporin activity, particularly PIP2;1 (Mondal and Molla 2025). Additional signaling elements including Ca^2+^ fluxes, ROS bursts, and antioxidant pathways are incorporated as plausible points of cross‐talk, as they commonly interact with ABA pathways and influence stomatal closure, root system architecture, and stress‐responsive gene expression across studies. The network also incorporates feedback loops and transcriptional cross‐talk, enabling cereals like wheat and barley to balance competing demands and maintain growth under resource‐limited conditions.

Beyond classical protein‐based signaling, studies show that cereals also rely on epigenetic and RNA‐mediated mechanisms to coordinate nitrogen and water responses. Genome‐wide methylation studies in maize and sorghum demonstrate that drought, low nitrogen, and osmotic stress can reshape CG and CHH methylation patterns at stress‐responsive loci, influencing transcriptional plasticity and root architecture (Rashid et al. 2022). These findings suggest that chromatin‐level regulation contributes to transcriptional flexibility and adjustment of stress‐response pathways under combined environmental constraints.

Non‐coding RNAs add a further regulatory tier. Several miRNAs known to target key transcription factors or nitrogen transport pathways respond strongly to water and nitrogen signals in cereals. For example, miR169 regulates NF‐YA activity during drought in maize and rice (Song et al. 2017), miR393 modulates auxin signaling and root architecture under nitrogen limitation in rice (Gao et al. 2011), and miR827 regulates nitrogen remobilization via NRT1/NPF transporters across multiple cereal species (Feng et al. 2014). Long non‐coding RNAs have also emerged as important integrators of stress signals: wheat lncRNAs modulate expression of NAC and WRKY factors during combined drought–nutrient stress (Zhang et al. 2022). In maize, small interfering RNAs (siRNAs) participate in maintaining chromatin stability under stress, contributing to epigenetic “memory” that can persist across developmental stages (Secco et al. 2015).

Together, these examples illustrate that chromatin dynamics and RNA‐based regulatory networks play a meaningful role in shaping how cereals balance nitrogen acquisition with water conservation. Although the integration of these mechanisms with ABA, calcium, and ROS signaling is still being clarified, they provide an important and increasingly recognized dimension of the coordinated regulation of NUE and WUE.

Breeding strategies can leverage these regulatory nodes by stacking favorable alleles, with the understanding that only a small subset of these genes has been evaluated beyond controlled environments, and most remain at the experimental validation stage. Importantly, while the model was developed using rice, maize, wheat, and barley, its modular design makes it broadly applicable to other cereals and potentially to diverse crop species.

### 4.1 Breeding Resilient Cereals: Harnessing Genomics for NUE and WUE Improvement

Securing the future of global cereal production demands a new generation of crops that excel in both nitrogen and water use efficiency. The presented genomic and network analyses confirm that the genetic drivers of these traits are not only deeply conserved, but also remarkably flexible. Across rice, wheat, barley, sorghum, and maize, gene families such as *NPF2.12* and *PIP2* persist as central players yet their chromosomal locations and regulatory connections shift in response to selection pressures and genomic rearrangements (Golicz et al. 2016).

Insights from the cross‐tolerance network model (Figure 4) and the comparative distribution of conserved gene families across cereal genomes (Figure 3) reinforce the idea that NUE and WUE are closely linked, with partial overlap in the regulatory pathways that shape their responses, particularly those involving ABA signaling, nitrate transport, and stress‐responsive transcription factors (Baillo et al. 2019; Reynolds and Langridge 2016). Wheat and barley carry distinctive variants, including *TaNPF2.12, HvNAC013*, and *TaPYL1‐1B*, that function at the intersection of nitrogen and drought stress responses (Mao et al. 2022; Siddiqui et al. 2023). Figure 2 offers a lens to understand how environmental constraints are translated into coordinated trait expression and gene regulation.

However, improving dual efficiency traits solely through single‐genotype selection can be limiting, as NUE and WUE are driven by multigenic, environment‐dependent responses that vary across sites, seasons, and management conditions.

This knowledge reframes the future of cereal breeding. True resilience must now be guided by targeted selection and powered by advanced breeding technologies. In this context, breeding strategies that maintain population‐level diversity offer meaningful advantages. Evolutionary breeding and dynamic mixture designs capitalize on population‐level diversity, promoting natural selection of genotypes with favorable trait combinations under variable field conditions (Döring et al. 2011; Litrico and Violle 2015). Evolutionary breeding involves growing genetically diverse populations over successive generations in target environments, allowing both natural and farmer‐mediated selection to enhance local adaptation without centralized control (Döring et al. 2011). Dynamic mixtures (also called variety mixtures or composite cross populations) are cultivated as heterogeneous stands, enabling ecological buffering and continuous adaptation to stress by maintaining genotypic diversity in the field (Litrico and Violle 2015). Participatory breeding, especially in smallholder contexts, aligns selection with real‐world stress exposures and farmer priorities (Ceccarelli and Grando 2007). This approach aligns cultivar development with local stress conditions, cultural practices, and farmer priorities, increasing the relevance, adoption, and resilience of breeding outcomes (Ceccarelli and Grando 2007).

Genomics‐based tools such as marker‐assisted selection and genomic prediction now enable precise stacking of desirable alleles. CRISPR/Cas genome editing accelerates this progress by directly targeting key loci like *NPF2.12* and *PIP2;1*, generating elite lines with improved stress responses (Wang et al. 2014; Zhang et al. 2018). Pan‐genomic resources and haplotype mining further expand the diversity available for breeding by revealing rare but impactful alleles (Varshney et al. 2021). High‐throughput phenotyping and multi‐omics platforms will ensure that these genetic gains are validated across both experimental and real‐world environments. Together, these innovations signal a shift from reactive breeding to proactive design enabling the construction of cereal ideotypes with integrated efficiencies. Although this study focuses on selected cereals, the regulatory frameworks and breeding strategies presented here provide a transferable blueprint for advancing complex traits across crop species. As climate and resource constraints intensify, uniting NUE and WUE within a breeding agenda is not only possible but imperative.

## Conflicts of Interest

The authors declare no conflicts of interest.

## Supporting information


**Supporting File:** pce70625‐sup‐0001‐Supplementary_Table_S1_Cereals.docx.

## Data Availability

This review includes figures and tables generated by the authors to synthesize and visualize existing literature. No new experimental data were produced. All sources referenced are publicly available and cited appropriately within the manuscript. No new datasets were generated in this study. All figures are based on published data and literature sources, which are fully cited in the manuscript. Conceptual diagrams and models were developed by the authors based on the reviewed literature.

## References

[pce70625-bib-0001] Ahmad, A. , Z. Aslam , T. Javed , et al. 2022. “Screening of Wheat (*Triticum aestivum* L.) Genotypes for Drought Tolerance Through Agronomic and Physiological Response.” Agronomy 12, no. 2: 287. 10.3390/agronomy12020287.

[pce70625-bib-0004] Araus, V. , J. Swift , J. M. Alvarez , A. Henry , and G. M. Coruzzi . 2020. “A Balancing Act: How Plants Integrate Nitrogen and Water Signals.” Journal of Experimental Botany 71, no. 15: 4442–4451. 10.1093/jxb/eraa054. 31990028 PMC7382378

[pce70625-bib-0005] Baillo, E. H. , R. N. Kimotho , Z. Zhang , and P. Xu . 2019. “Transcription Factors Associated With Abiotic and Biotic Stress Tolerance and Their Potential for Crops Improvement.” Genes 10, no. 10: 771. 10.3390/genes10100771. 31575043 PMC6827364

[pce70625-bib-0006] Bardehji, S. , H. R. Eshghizadeh , M. Zahedi , M. R. Sabzalian , and M. Gheisari . 2021. “The Combined Effect of Nitrogen Fertilizer and Sowing Season on Response to Water‐Limited Stress in Barley (Hordeum vulgare L).” Journal of Agricultural Science 159, no. 1–2: 31–49. 10.1017/S0021859621000149.

[pce70625-bib-0007] Blum, A. 2009. “Effective Use of Water (EUW) and Not Water‐Use Efficiency (WUE) Is the Target of Crop Yield Improvement Under Drought Stress.” Field Crops Research 112, no. 2–3: 119–123. 10.1016/j.fcr.2009.03.009.

[pce70625-bib-0008] Borrell, A. K. , and G. L. Hammer . 2000. “Nitrogen Dynamics and the Physiological Basis of Stay‐Green in Sorghum.” Crop Science 40, no. 5: 1295–1307. 10.2135/cropsci2000.4051295x.

[pce70625-bib-0009] Cai, Y. , Y. Wang , Y. Li , Y. Liu , and J. Cao . 2023. “Functional Characterization of ZmGS3 and Its Role in Post‐Anthesis Nitrogen Remobilization in Maize.” Plant Physiology and Biochemistry 194: 62–71. 10.1016/j.plaphy.2023.107366.

[pce70625-bib-0010] Caine, R. S. , X. Yin , J. Sloan , et al. 2019. “Rice With Reduced Stomatal Density Conserves Water and Has Improved Drought Tolerance under Future Climate Conditions.” New Phytologist 221, no. 1: 371–384. 10.1111/nph.15344. 30043395 PMC6492113

[pce70625-bib-0011] Ceccarelli, S. , and S. Grando . 2007. “Decentralized‐Participatory Plant Breeding: An Example of Demand‐Driven Research.” Euphytica 155, no. 3: 349–360. 10.1007/s10681-007-9381-2.

[pce70625-bib-0012] Ciampitti, I. A. , and T. J. Vyn . 2014. “Understanding Global and Historical Nutrient Use Efficiencies for Closing Maize Yield Gaps.” Agronomy Journal 106, no. 6: 2107–2117. 10.2134/agronj14.0025.

[pce70625-bib-0101] Congreves, K. A. , and L. L. Van Erd . 2015. “Nitrogen Cycling and Management in Intensive Horticultural Systems.” Nutrient Cycling in Agroecosystems 102: 299–318. 10.1007/s10705-015-9704-7.

[pce70625-bib-0014] Cutler, S. R. , P. L. Rodriguez , R. R. Finkelstein , and S. R. Abrams . 2010. “Abscisic Acid: Emergence of a Core Signaling Network.” Annual Review of Plant Biology 61, no. 1: 651–679. 10.1146/annurev-arplant-042809-112122. 20192755

[pce70625-bib-0016] Döring, T. F. , S. Knapp , G. Kovacs , K. Murphy , and M. S. Wolfe . 2011. “Evolutionary Plant Breeding in Cereals—Into a New Era.” Sustainability 3, no. 10: 1944–1971. 10.3390/su3101944.

[pce70625-bib-0017] Duma, S. , H. Shimelis , and T. J. Tsilo . 2022. “Response of Bread Wheat Genotypes for Drought and Low Nitrogen Stress Tolerance.” Agronomy 12, no. 6: 1384. 10.3390/agronomy12061384.

[pce70625-bib-0018] Faber, A. , L. Zimny , and M. Liszewska . 2016. “Analysis of Water and Nitrogen Use Efficiency in Winter Wheat Grown Under Mildly Dry Conditions.” Polish Journal of Agronomy 26: 23–27.

[pce70625-bib-0019] Fan, X. , Z. Tang , Y. Tan , et al. 2016. “Overexpression of a pH‐Sensitive Nitrate Transporter in Rice Increases Crop Yields.” Proceedings of the National Academy of Sciences 113, no. 26: 7118–7123. 10.1073/pnas.1525184113. PMC4932942 27274069

[pce70625-bib-0020] Feng, H. , M. Xu , W. Liu , L. Xiong , G. Xu , and Z. He . 2014. “Nutrient‐Responsive microRNAs in Maize: Roles in Nitrogen Homeostasis.” Journal of Integrative Plant Biology 56, no. 11: 1060–1070. 10.1111/jipb.12178.

[pce70625-bib-0021] Food and Agriculture Organization of the United Nations . 2021. *FAOSTAT statistical database*. https://www.fao.org/faostat/en/.

[pce70625-bib-0022] Food and Agriculture Organization of the United Nations . 2023. *The state of food and agriculture 2023: Revealing the true cost of food to transform agrifood systems*. 10.4060/cc8166en.

[pce70625-bib-0102] Gao, P. , X. Bai , L. Yang , et al. 2011. “Osa‐MIR393: A Salinity‐ and Alkaline Stress‐Related MicroRNA Gene.” Molecular Biology Reports 38, no. 1: 237–242. 10.1007/s11033-010-0100-8. 20336383

[pce70625-bib-0025] Golicz, A. A. , P. E. Bayer , G. C. Barker , et al. 2016. “The Pangenome of an Agronomically Important Crop Plant *Brassica oleracea* .” Nature Communications 7: 13390. 10.1105/tpc.16.00488. PMC5114598 27834372

[pce70625-bib-0103] Gong, Z. , L. Xiong , H. Shi , et al. 2020. “Plant Abiotic Stress Response and Nutrient Use Efficiency.” Science China Life Sciences. 10.1007/s11427-020-1683-x. 32246404

[pce70625-bib-0027] Govta, N. , L. Govta , H. Sela , et al. 2025. “Plasticity of Root System Architecture and Whole Transcriptome Responses Underlying Nitrogen Deficiency Tolerance Conferred by a Wild Emmer Wheat QTL.” Plant, Cell & Environment 48, no. 4: 2835–2855. 10.1111/pce.15416. PMC11893928 39887777

[pce70625-bib-0104] He, X. , B. Qu , W. Li , et al. 2015. “The Nitrate‐Inducible NAC Transcription Fcator TaNAC2‐5a Controls Nitrate Response and Increases Wheat Yield.” Plant Physiology 169, no. 3: 1991–2005. 10.1104/pp.15.00568. 26371233 PMC4634051

[pce70625-bib-0029] Hodapp, D. , H. Hillebrand , and M. Striebel . 2019. “Unifying” the Concept of Resource Use Efficiency in Ecology.” Frontiers in Ecology and Evolution 6: 2. 10.3389/fevo.2018.00233.

[pce70625-bib-0105] Hove, R. M. , M. Ziemann , and M. Bhave . 2015. “Identification and Expression Analysis of the Barley (*Hordeum vulgare L*.) Aquaporin Gene Family.” PLOS ONE 10, no. 6: e0128025. 10.1371/journal.pone.0128025. 26057533 PMC4461243

[pce70625-bib-0031] Hu, B. , W. Wang , S. Ou , et al. 2015. “Variation in NRT1.1B Contributes to Nitrate‐Use Divergence Between Rice Subspecies.” Nature Genetics 47, no. 7: 834–838. 10.1038/ng.3337. 26053497

[pce70625-bib-0032] Hu, W. , D. A. Loka , Y. Yang , et al. 2024. “Partial Root‐Zone Drying Irrigation Improves Intrinsic Water‐Use Efficiency and Maintains High Photosynthesis by Uncoupling Stomatal and Mesophyll Conductance in Cotton Leaves.” Plant, Cell & Environment 47, no. 8: 3147–3165. 10.1111/pce.14932. 38693776

[pce70625-bib-0033] International Energy Agency . 2021. *Net Zero by 2050: A Roadmap for the Global Energy Sector*. https://www.iea.org/reports/net-zero-by-2050.

[pce70625-bib-0106] Jia, Y. , D. Qin , Y. Zheng , and Y. Wang . 2023. “Finding Balance in Adveristy. Nitrate Signaling as the key Toplant Growth, Resilience, and Stress Resposne.” International Journal of Molecular Sciences 24, no. 19: 14406. 10.3390/ijms241914406. 37833854 PMC10572113

[pce70625-bib-0107] Jing, Y. , C. Shen , and W. Li . 2024. “TaLBD41 Interacts With TaNAC2 to Regulate Nitrogen Uptake and Metabolism in Response to Nitrate Availability.” New Phytologist 242: 641–657. 10.1111/nph.19579. 38379453

[pce70625-bib-0035] Kamara, A. Y. , S. U. Ewansiha , and A. Menkir . 2014. “Assessment of Nitrogen Uptake and Utilization in Drought Tolerant and *Striga* Resistant Tropical Maize Varieties.” Archives of Agronomy and Soil Science 60, no. 2: 195–207. 10.1080/03650340.2013.783204.

[pce70625-bib-0036] Knipfer, T. , and W. Fricke . 2020. “Stomatal Behaviour and Water Transport in Plants under Stress.” Plant Physiology 183, no. 1: 49–66. 10.1104/pp.20.00500.

[pce70625-bib-0037] Koua, A. P. , B. C. Oyiga , M. M. Baig , J. Léon , and A. Ballvora . 2021. “Breeding Driven Enrichment of Genetic Variation for Key Yield Components and Grain Starch Content Under Drought Stress in Winter Wheat.” Frontiers in Plant Science 12: 684205. 10.3389/fpls.2021.684205. 34484257 PMC8415485

[pce70625-bib-0038] Kulkarni, M. , R. Soolanayakanahally , S. Ogawa , Y. Uga , M. G. Selvaraj , and S. Kagale . 2017. “Drought Response in Wheat: Key Genes and Regulatory Mechanisms Controlling Root System Architecture and Transpiration Efficiency.” Frontiers in Chemistry 5: 113. 10.3389/fchem.2017.00106. 29259968 PMC5723305

[pce70625-bib-0041] Li, T. , B. Li , Y. Wang , et al. 2025. “WRKY Transcription Factors in Rice: Key Regulators Orchestrating Development and Stress Resilience.” Plant, Cell & Environment 48, no. 11: 8388–8406. 10.1111/pce.70124. 40831341

[pce70625-bib-0108] Li, Y. , J. Ouyang , Y.‐Y. Wang , et al. 2015. “Disruption of the Rice Nitrate Transporter OsNPF.2 Hinders Root‐to‐Shoot Nitrate Transport and Vascular Develpoment.” Scientific Reports 5: 9635. 10.1038/srep09635. 25923512 PMC5386202

[pce70625-bib-0109] Li, Q. , X. Yang , G. Bai , et al. 2010. “Cloning and Characterization of a Putative GS3 Ortholog Involved in Maize Kernel Development.” Theoretical and Applied Genetics 120: 753–763. 10.1007/s00122-009-1196-x. 19898828

[pce70625-bib-0042] Litrico, I. , and C. Violle . 2015. “Diversity in Plant Breeding: A New Conceptual Framework.” Trends in Plant Science 20, no. 10: 604–613. 10.1016/j.tplants.2015.07.007. 26440430

[pce70625-bib-0043] Liu, H. , S. Song , M. Liu , et al. 2023. “Transcription Factor ZmNAC20 Improves Drought Resistance by Promoting Stomatal Closure and Activating Expression of Stress‐Responsive Genes in Maize.” International Journal of Molecular Sciences 24, no. 5: 4712. 10.3390/ijms24054712. 36902144 PMC10003513

[pce70625-bib-0045] Liu, Z. , J. Gao , S. Zhao , et al. 2023. “Nitrogen Responsiveness of Leaf Growth, Radiation Use Efficiency, and Grain Yield of Maize (*Zea mays* L.) in Northeast China.” Field Crops Research 291: 108806. 10.1016/j.fcr.2022.108806.

[pce70625-bib-0110] Lopes, M. S. , and M. P. Reynolds . 2010. “Partitioning of Assimilates to Deeper Roots is Associated With Cooler Canopies and Increased Yield Under Drought in Wheat.” Functional Plant Biology 37: 147–156. 10.1071/FPO9121.

[pce70625-bib-0048] Lynch, J. P. 2019. “Root Phenotypes for Improved Nutrient Capture: An Underexploited Opportunity for Global Agriculture.” New Phytologist 223, no. 4: 548–564. 10.1111/nph.15738. 30746704

[pce70625-bib-0050] Mao, H. , C. Jian , X. Cheng , et al. 2022. “The Wheat ABA Receptor Gene TaPYL1‐1B Contributes to Drought Tolerance and Grain Yield by Increasing Water‐Use Efficiency.” Plant Biotechnology Journal 20, no. 5: 846–861. 10.1111/pbi.13764. 34890091 PMC9055818

[pce70625-bib-0111] Mao, C. , S. Lu , B. Lv , et al. 2017. “A Rice NAC Trancription Factor Promotes Leaf sensescence via ABA Biosynthesis.” Plant Physiology 174, no. 3: 1747–1763. 10.1104/pp.17.00542. 28500268 PMC5490923

[pce70625-bib-0051] Marchive, C. , F. Roudier , and L. Castaings , et al. 2013. “Nuclear Retention of the Transcription Factor NLP7 Orchestrates the Early Response to Nitrate in Plants.” Nature Communications 4: 1713. 10.1038/ncomms2650. 23591880

[pce70625-bib-0052] Maurel, C. , Y. Boursiac , D. T. Luu , V. Santoni , Z. Shahzad , and L. Verdoucq . 2015. “Aquaporins in Plants.” Physiological Reviews 95, no. 4: 1321–1358. 10.1152/physrev.00008.2015. 26336033

[pce70625-bib-0053] Medrano, H. , M. Tomás , S. Martorell , et al. 2015. “From Leaf to Whole‐Plant Water Use Efficiency (WUE) in Complex Canopies: Limitations of Leaf WUE as a Selection Target.” Crop Journal 9, no. 4: 758–769. 10.1016/j.cj.2015.04.002.

[pce70625-bib-0054] Meng, X. , H. Lou , S. Zhai , et al. 2024. “TaNAM‐6A Is Essential for Nitrogen Remobilisation and Regulates Grain Protein Content in Wheat (*Triticum aestivum* L.).” Plant, Cell & Environment 47, no. 6: 2310–2321. 10.1111/pce.14878. 38494960

[pce70625-bib-0055] Moll, R. H. , E. J. Kamprath , and W. A. Jackson . 1982. “Analysis and Interpretation of Factors Which Contribute to Efficiency of Nitrogen Utilization.” Agronomy Journal 74, no. 3: 562–564. 10.2134/agronj1982.00021962007400030037x.

[pce70625-bib-0112] Mondal, R. , and K. A. Molla . 2025. “DREB1C: Connecting Dots Between Water‐ and Nitrogen‐Use Efficiency to Climate‐Smart Crop Development.” Journal of Plant Growth Regulation 44: 1389–1395. 10.1007/s00344-023-11160-3.

[pce70625-bib-0057] Morris, G. P. , P. Ramu , S. P. Deshpande , et al. 2013. “Population Genomic and Genome‐Wide Association Studies of Agroclimatic Traits in Sorghum.” Proceedings of the National Academy of Sciences 110, no. 2: 453–458. 10.1073/pnas.1215985110. PMC3545811 23267105

[pce70625-bib-0058] Munns, R. , R. A. James , B. Xu , et al. 2012. “Wheat Grain Yield on Saline Soils Is Improved by an Ancestral Na^+^ Transporter Gene.” Nature Biotechnology 30, no. 4: 360–364. 10.1038/nbt.2120. 22407351

[pce70625-bib-0059] Ochieng, G. , K. Ngugi , L. N. Wamalwa , et al. 2021. “Novel Sources of Drought Tolerance From Landraces and Wild Sorghum Relatives.” Crop Science 61, no. 1: 104–118. 10.1002/csc2.20300.

[pce70625-bib-0113] Oyiga, B. C. , J. Palczak , T. Wojciechowski , et al. 2020. “Genetic Components Ofroot Architecture and Anatomy Adjustements to Water‐Deficit Stress in Spring Barley.” Plant, Cell & Environment 43, no. 5: 11192–11207. 10.1111/pce.13683. 31734943

[pce70625-bib-0061] Plett, D. C. , K. Ranathunge , V. J. Melino , N. Kuya , Y. Uga , and H. J. Kronzucker . 2020. “The Intersection of Nitrogen Nutrition and Water Use in Plants: New Paths Toward Improved Crop Productivity.” Journal of Experimental Botany 71, no. 15: 4452–4468. 10.1093/jxb/eraa049. 32026944 PMC7382376

[pce70625-bib-0114] Rashid, M. , A. Vaishnav , R. K. Verma , et al. 2022. “Epigenetic Regulation of Salinity Stress Responses in Cereals.” Molecular Biology Reports 49: 761–772. 10.1007/s11033-021-06922-9. 34773178

[pce70625-bib-0063] Reynolds, M. , and P. Langridge . 2016. “Physiological Breeding.” Current Opinion in Plant Biology 31: 162–171. 10.1016/j.pbi.2016.04.005. 27161822

[pce70625-bib-0064] Sack, L. , and C. Scoffoni . 2013. “Leaf Venation: Structure, Function, Development, Evolution, Ecology and Applications in the Past, Present and Future.” New Phytologist 198, no. 4: 983–1000. 10.1111/nph.12253. 23600478

[pce70625-bib-0065] Sadras, V. O. , and D. Rodriguez . 2010. “Modelling the Nitrogen‐Driven Trade‐Off Between Nitrogen Utilisation Efficiency and Water Use Efficiency of Wheat in Eastern Australia.” Field Crops Research 118, no. 3: 297–305. 10.1016/j.fcr.2010.06.010.

[pce70625-bib-0066] Saengwilai, P. , E. A. Nord , J. G. Chimungu , K. M. Brown , and J. P. Lynch . 2014. “Root Cortical Aerenchyma Enhances Nitrogen Acquisition From Low‐Nitrogen Soils in Maize.” Plant Physiology 166, no. 2: 726–735. https://academic.oup.com/plphs/article-abstract/166/2/726/6113319. 24891611 10.1104/pp.114.241711 PMC4213101

[pce70625-bib-0067] Samant, S. B. , J. Swain , N. Yadav , et al. 2025. “Overexpression of Phytoglobin1 in Rice Leads to Enhanced Nitrogen Use Efficiency via Modulation of Nitric Oxide.” Plant, Cell & Environment 48, no. 4: 2755–2768. 10.1111/pce.15289. 39569580

[pce70625-bib-0068] Schneider, H. M. , and J. P. Lynch . 2020. “Should Root Plasticity be a Crop Breeding Target?” Frontiers in Plant Science 11: 546. 10.3389/fpls.2020.00546. 32499798 PMC7243933

[pce70625-bib-0069] Secco, D. , C. Wang , H. Shou , et al. 2015. “Stress‐Induced Gene Expression Drives Transient DNA Methylation Changes at Adjacent Repetitive Elements.” eLife 4: e09343. 10.7554/eLife.09343. 26196146 PMC4534844

[pce70625-bib-0070] Seleiman, M. F. , N. Al‐Suhaibani , N. Ali , et al. 2021. “Drought Stress Impacts on Plants and Different Approaches to Alleviate Its Adverse Effects.” Plants 10, no. 2: 259. 10.3390/plants10020259. 33525688 PMC7911879

[pce70625-bib-0071] Sharma, L. , and S. Bali . 2018. “A Review of Methods to Improve Nitrogen Use Efficiency in Agriculture.” Sustainability 10, no. 1: 51. 10.3390/su10010051.

[pce70625-bib-0115] Siddiqui, M. N. , K. Pandey , S. K. Bhadhury , et al. 2023. “Convergently Selected NPF2.12 Coordinates Root Growth and Nitrogen Use Efficiency in Wheat and Barley.” New Phytologist 238: 2175–2193. 10.1111/nph.18820. 36808608

[pce70625-bib-0073] Sinclair, T. R. 2011. “Challenges in Breeding for Yield Increase for Drought.” Trends in Plant Science 16, no. 6: 289–293. 10.1016/j.tplants.2011.02.008. 21419688

[pce70625-bib-0074] Soma, F. , F. Takahashi , K. Yamaguchi‐Shinozaki , and K. Shinozaki . 2021. “Cellular Phosphorylation Signaling and Gene Expression in Drought Stress Responses: ABA‐Dependent and ABA‐Independent Regulatory Systems.” Plants 10, no. 4: 756. 10.3390/plants10040756. 33924307 PMC8068880

[pce70625-bib-0075] Song, Q. X. , Y. F. Liu , and X. J. Hu , et al. 2017. “Identification of Drought‐Responsive microRNAs in Maize.” Scientific Reports 7: 108. 10.1038/s41598-017-00140-6.28273897

[pce70625-bib-0076] Tabuchi, M. , K. Sugiyama , K. Ishiyama , et al. 2005. “Severe Reduction in Growth Rate and Grain Filling of Rice Mutants Lacking OsGS1;1, a Cytosolic Glutamine Synthetase1;1.” Plant Journal : For Cell and Molecular Biology 42, no. 5: 641–651. 10.1111/j.1365-313X.2005.02406.x. 15918879

[pce70625-bib-0077] Tardieu, F. 2012. “Any Trait or Trait‐Related Allele Can Confer Drought Tolerance: Just Design the Right Drought Scenario.” Journal of Experimental Botany 63, no. 1: 25–31. 10.1093/jxb/err269. 21963615

[pce70625-bib-0079] Thomas, H. , and H. Ougham . 2014. “The Stay‐Green Trait.” Journal of Experimental Botany. Journal of Experimental Botany 65, no. 14: 3889–3900. 10.1093/jxb/eru169. 24600017

[pce70625-bib-0080] Tolley, S. , and M. Mohammadi . 2020. “Variation in Root and Shoot Growth in Response to Reduced Nitrogen.” Plants 9, no. 2: 144. 10.3390/plants9020144. 31979237 PMC7076707

[pce70625-bib-0081] Uga, Y. , K. Sugimoto , S. Ogawa , et al. 2013. “Control of Root System Architecture by Deeper Rooting 1 Increases Rice Yield under Drought Conditions.” Nature Genetics 45, no. 9: 1097–1102. 10.1038/ng.2725. 23913002

[pce70625-bib-0083] Varshney, R. K. , R. Barmukh , M. Roorkiwal , et al. 2021. “Breeding Custom‐Designed Crops for Improved Drought Adaptation.” Advanced Genetics (Hoboken, N.J.) 2, no. 9: e202100017. 10.1002/ggn2.202100017. PMC9744523 36620433

[pce70625-bib-0084] Wang, D. , S. Liu , M. Guo , et al. 2025. “Optimizing Nitrogen Fertilization and Irrigation Practices for Enhanced Winter Wheat Productivity in the North China Plain: A Meta‐Analysis.” Plants 14, no. 11: 1686. 10.3390/plants14111686. 40508360 PMC12157333

[pce70625-bib-0086] Wang, Y. , X. Cheng , Q. Shan , et al. 2014. “Simultaneous Editing of Three Homoeoalleles in Hexaploid Bread Wheat Confers Heritable Resistance to Powdery Mildew.” Nature Biotechnology 32, no.9: 947–951. 10.1038/nbt.2969. 25038773

[pce70625-bib-0088] World Resources Institute . 2023. Aqueduct Water Risk Atlas. World Resources Institute. https://www.wri.org/applications/aqueduct/water-risk-atlas.

[pce70625-bib-0116] Xin, L. 2022. “Chemical Fertilizer Rate, use Efficiency and Reduction of Cereal Crops in China, 1998‐2018.” Journal of Geographical Sciences 32: 65–78. 10.1007/s11442-022-1936-2.

[pce70625-bib-0089] Yang, J. T. , H. M. Schneider , K. M. Brown , and J. P. Lynch . 2019. “Genotypic Variation and Nitrogen Stress Effects on Root Anatomy in Maize Are Node Specific.” Journal of Experimental Botany 70, no. 19: 5311–5325. 10.1093/jxb/erz293. 31231768 PMC6793441

[pce70625-bib-0118] Zhang, Y. , C. Chen , X.‐F. Jin , et al. 2009. “Expression of a Rice DREB! Gene, OsDREB!D; Enhamces Cold and High‐Salt Tolerance in Transgenic Arabidopsis.” BMB Reports 42, no. 8: 486–492. 10.5483/BMBRep.2009.42.8.486. 19712584

[pce70625-bib-0117] Zhang, Y. , S. Li , S. Xue , et al. 2018. “Phylogenetic and CRISPR/Cas9 Studies in Deciphering the Evolutionary Trajectory and Phenotypic Impacts of Rice ERECTA Genes.” Frontiers in Plant Science 9: 473. 10.3389/fpls.2018.00473. 29692796 PMC5902711

[pce70625-bib-0091] Zhang, Y. , K. Massel , I. D. Godwin , and C. Gao . 2018. “Applications and Potential of Genome Editing in Crop Improvement.” Genome Biology 21: 210. 10.1186/s13059-018-1586-y. PMC6267055 30501614

[pce70625-bib-0092] Zhang, Y. , L. Song , G. Li , and Y. Song . 2022. “Interplay of Nitrogen and Water Availability on Photosynthesis and Grain Yield in Cereals: Mechanisms and Management.” Plant Physiology and Biochemistry 180: 71–82. 10.1016/j.plaphy.2022.02.001.

[pce70625-bib-0120] Zhang, X. , J. Zhang , L. Li , Y. Liu , W. Zhen , and G. Wang . 2024. “Interaction Effects of Water and Nitrogen Practices on Wheat Yield, Water and Nitrogen Productivity Under Drip Fertigation in Northern China.” Agriculture 14, no. 9: 1496. 10.3390/agriculture14091496.

[pce70625-bib-0119] Zhou, X. , D. Yi , L. Ma , and X. Wang . 2024. “Genome‐Wide Analysis and Expression of the Aquaporin Gene Family in *Avena sativa L* .” Frontiers in Plant Science 14: 1305299. 10.3389/fpls.2023.1305299. 38312362 PMC10836146

[pce70625-bib-0095] Zhu, J.‐K. 2016. “Abiotic Stress Signaling and Responses in Plants.” Cell 167, no. 2: 313–324. 10.1016/j.cell.2016.08.029. 27716505 PMC5104190

