# Supplementary material for: Breeding for Resilient Cereals: Integrating Molecular Networks and Trait‐Based Selection to Improve Nitrogen and Water Use Efficiencies
Source: Plant Cell Environ. 2026 Jun 2;49(9):6583–95. doi: 10.1111/pce.70625 (PMC13436516; doi:10.1111/pce.70625)
Supplement: Supplementary file 1 [file PCE-49-6583-s001.docx]

# Supplementary Materials

## Table S1. Key Genetic Loci and Candidate Genes Associated with Nitrogen and Water Use Efficiency in Cereals

Table S1 presents a consolidated view of the key genes and mapped loci associated with water and nitrogen use efficiency in cereals. This synthesis includes chromosomal locations, functional roles, and supporting references. Highlighted genes are involved in processes such as stomatal regulation, root water uptake, nitrate transport, and nitrogen assimilation, offering promising targets for breeding and genome editing strategies across major cereal species.

| Trait Focus | Crop | Gene / Locus | Chromosome | Function / Trait | Reference |
| --- | --- | --- | --- | --- | --- |
| NUE | Rice | *OsNPF2.2* | Chr 6 | Nitrate translocation | Li et al., 2015 |
| NUE | Rice | *OsNPF6.5 (NRT1.1B)* | Chr 4 | Allelic control of uptake and transport | Hu et al., 2015 |
| NUE | Wheat | *NPF2.12* homolog | Unknown | Deeper rooting and nitrate uptake | Siddiqui et al., 2023 |
| NUE | Barley | *HvGS1-1* | Chr 6H | GS activity and grain protein | Gao et al., 2019 |
| NUE | Sorghum | *SbNPF6.4* | Chr 6 | Shoot nitrate content | Morris et al., 2013 |
| NUE | Sorghum | *SbNRT2.5* | Chr 2 | High-affinity nitrate uptake | Zheng et al., 2020 |
| NUE | Maize | *ZmGS3* | Chr 3 | Post-anthesis N efficiency | Cai et al., 2023 |
| NUE | Foxtail Millet | *SiWRKY / SiDof / SiNAC* | Various | Transcriptional control of NUE | Wang et al., 2021 |
| NUE | Pearl Millet | *QTL* for root vigor | Chr 3 | Early vigor and deep rooting | Yadav et al., 2022 |
| NUE | Teff | Putative nitrate transporters | Unknown | Transporter upregulation | Ayele et al., 2021 |
| WUE | Rice | *OsEPF1* | Chr 6 | Reduced stomatal density | Caine et al., 2019 |
| WUE | Wheat | *ERECTA* | Chr 2 | Epidermal patterning | Dunn et al., 2019 |
| WUE | Barley | Stomatal density QTL | Field mapped | Lower transpiration rate | Głowacka et al., 2018 |
| WUE | Maize | *HT1 / SLAC1 / OST1 / GHR1* | Various | Dynamic stomatal response | Drake et al., 2013 |
| WUE | Maize | *ZmTIP2;2* | Chr 5 | Turgor maintenance | Zhang et al., 2021 |
| WUE | Rice | *OsPIP2;5* | Chr 4 | Root water transport | Lian et al., 2021 |
| WUE | Wheat | *TaPYL1-1B* | Chr 1B | ABA-mediated stomatal closure | Mao et al., 2022 |
| WUE | Wheat | *δ13C* QTLs | 1B, 3A, 4D | Canopy cooling and transpiration efficiency | Siddiqui et al., 2023 |

Note: Genes marked as validated are supported by functional characterization or transgenic experiments, while others are based on mapped QTLs or expression evidence pending direct confirmation.
